# Child-Witnessed Domestic Violence and its Adverse Effects on Brain Development: A Call for Societal Self-Examination and Awareness

**DOI:** 10.3389/fpubh.2014.00178

**Published:** 2014-10-10

**Authors:** Areti Tsavoussis, Stanislaw P. A. Stawicki, Nicoleta Stoicea, Thomas J. Papadimos

**Affiliations:** ^1^Lucas County Prosecutor’s Office, Victim Witness Assistance Program, Toledo, OH, USA; ^2^Department of Research and Innovation, St. Luke’s University Health Network, Temple University School of Medicine, Bethlehem, PA, USA; ^3^Department of Anesthesiology, The Ohio State University Wexner Medical Center, Columbus, OH, USA

**Keywords:** central nervous system, child abuse, domestic violence, endocrine system, Ohio, pediatrics, philosophy, post-traumatic stress disorder

## Abstract

There is substantial evidence indicating that children who witness domestic violence (DV) have psychosocial maladaptation that is associated with demonstrable changes in the anatomic and physiological make up of their central nervous system. Individuals with these changes do not function well in society and present communities with serious medical, sociological, and economic dilemmas. In this focused perspective, we discuss the psychosocially induced biological alterations (midbrain, cerebral cortex, limbic system, corpus callosum, cerebellum, and the hypothalamic, pituitary, and adrenal axis) that are related to maladaptation (especially post-traumatic stress disorder) in the context of child-witnessed DV, and provide evidence for these physical alterations to the brain. Herein, we hope to stimulate the necessary political discourse to encourage legal systems around the world to make the act of DV in the presence of a child, including a first time act, a stand-alone felony.

## Background

Domestic violence (DV) is a devastating problem that affects individuals around the world. Data collected in 2001–2005 from a study of non-institutionalized adults in the USA – National Epidemiologic Survey on Alcohol and Related Conditions (NESARC) – indicated that emotional neglect was the most common childhood reported maltreatment with a prevalence of 3.4–9.2% (1). A recent meta-analysis has raised concerns over child neglect as an extensive problem with severe consequences requiring future prevalence studies in low-resource countries (2).

While our perspective is from the vantage point of the USA, specifically Ohio, DV is without a doubt an international problem (2, 3). This brief perspective serves to raise the level of awareness in regard to deleterious effects that occur in children who witness DV. We would like for our readers to understand that witnessing DV (let alone being the primary target) makes children susceptible to post-traumatic stress disorder (PTSD) (4, 5). Such an exposure may result in anatomical and physiological alterations in their brain structure, with subsequent personal and social consequences (6, 7). Schwab-Stone et al. indicate that there is a relationship between a history of childhood maltreatment and an internalizing of the symptoms of anxiety, depression, somatization, and externalizing antisocial behavior; these findings did not demonstrate sex or ethnic differences (8).

**Figure 1:**
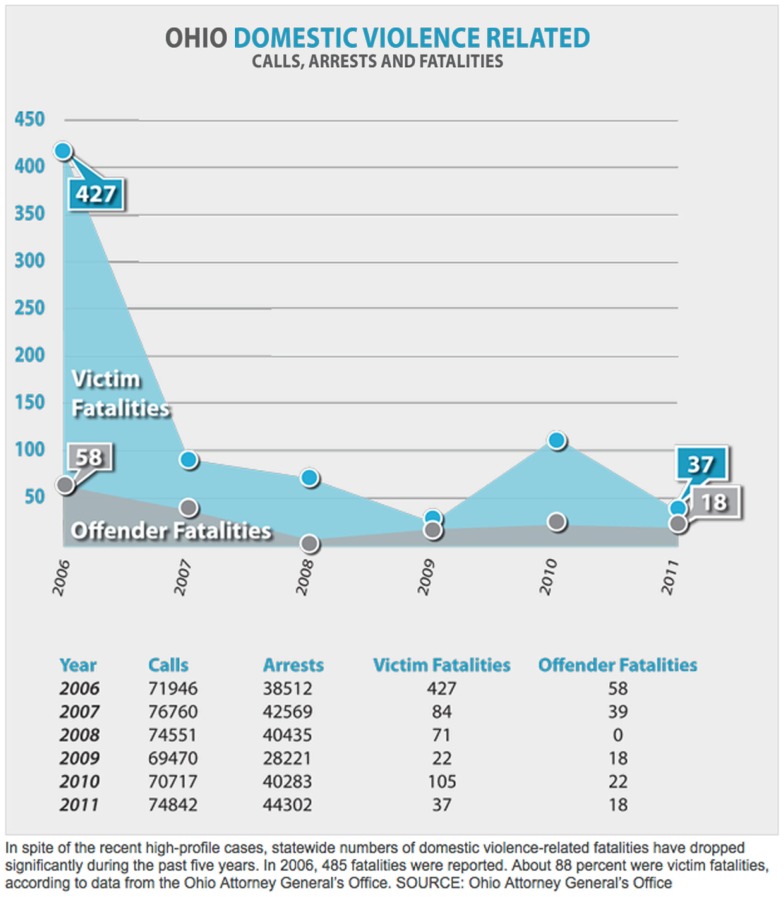
**Ohio domestic related violence facts 2006–2011**.

The problem of DV in Ohio from 2006 to 2011 was concerning, with approximately 70,000–75,000 total calls to authorities resulting in 40,000–45,000 arrests; 15,000–20,000 children were served in this process (9). However, over this period of time (2006–2011) the number of fatalities due to DV fell considerably (Figure 1), secondary to state wide interventions through regulations, laws, and enforcement of applicable laws. Such success in Ohio can be attributed to a variety of efforts and interventions. Generally, there has been an increase in the provision of legal services to victims, an improving educational and economic environment for women, and the changing demographic trends of an aging population with an accompanied increase in racial diversity (10). Most importantly, multiple revisions of Ohio Regulatory Code 2919.25 that was originally passed in 2003, with revisions in 2009 and 2010, have been very helpful (11). Essentially, these revisions established the third act of DV as a third degree felony (felonies in Ohio are rated 1–5, with 1 being the worst), although for the first two offenses the offender may be charged with only a misdemeanor. However, if the person attacked was a family or household member it becomes a fourth degree felony, and if the victim was pregnant, a mandatory prison term is imposed. Of further note, as of March 22, 2014 (Ohio Senate Bill 160 passed originally in 2012) Ohio judges can now sentence those who commit fourth and fifth degree felonies, the lowest felony levels, to prison for a first time offense (12). The new legislation means judges can now directly sentence an offender with a fourth or fifth degree felony to prison on a first offense if the crime involves a firearm, physical harm, a bail or bond violation, a violation of a community sanction imposed earlier, any sexual offense, any criminal act done for hire, or if the offender abused certain positions of trust such as in public office or law enforcement. Also, news briefs and interventions supported by the Ohio Children’s Defense Fund, especially to children who observe DV, have provided a positive impact (13).

Three bills under consideration at this time in Ohio offer greater protections to DV victims and give them more legal protection in regard to their employment, housing, and economic livelihood. The bills are House Bill (H.B.) 243, H.B. 160, and H.B. 297 and would require individuals served with temporary protection orders to surrender their firearms (H.B. 243) and offer legal protection to the pets of DV victims, often given as a reason for a victim’s inability to leave a violent environment (H.B.160). Of greatest importance are the changes that would be implemented by H.B. 297. This bill outlines new legal protections for DV victims who need to terminate a rental agreement or take unpaid leave at work in order to deal with DV incidents. Under this bill, DV victims would have legal protection against termination of their job and would have the option to dissolve a rental lease if they have been victimized. The bill would also stop landlords from evicting tenants who have been victims of DV at their residence, and requires the landlords to comply with requests to change locks when a tenant has been stalked or threatened (14), see Table 1.

**Table 1:** **Important interventions/demographic changes that abated Ohio domestic violence**.

Social/economic	Improving economic opportunities for women; aging population.
Organizational	Ohio Domestic Violence Network; Ohio Child Defense Fund Interventions, conferences, grants, and distribution of literature.
Legal	Increase in the provision of legal services; changes in Ohio Revised Code (2003, 2007, 2009, 2010) for easier and more severe sentencing of offenders. Also, pending new legislation supportive of domestic violence victims’ personal environment (see text).

In the subsequent sections, we outline the up-to-date understanding of the connection between the child-witnessing DV and the potential for the emergence of PTSD, including long-term sequelae. We use this perspective to argue for child-witnessed DV to carry a felony charge on the first offense.

## Discussion

### Post-traumatic stress disorder defined

The criteria for PTSD have changed in the Diagnostic and Statistical Manual (DSM) 5 of May 2013 (15), see Table S1 in Supplementary Material. PTSD criteria have now been divided into sections for those over 6 years of age and those 6 years of age and under (for symptom duration of more than one month). In the older group, PTSD encompasses the following: (1) exposure to actual or threatened death, serious injury, or sexual violence, (2) the presence of intrusion symptoms associated with the traumatic event(s) (repetitive play themes, distressing dreams, flashbacks, physiological reactions, marked psychological distress), (3) persistent avoidance of stimuli associated with the traumatic event(s), (4) negative alterations in cognition and mood associated with the event, and (5) marked alterations in arousal associated with the event(s) (irritability, anger, recklessness, hypervigilance, exaggerated startle response, problems with concentration, and sleep disturbances) (15). Among those less than 6 years of age directly experiencing the trauma, witnessing the events as they occurred, especially to primary caregivers, or merely learning that an event had occurred to a parent or primary caregiver will be enough to trigger PTSD (15). Also, it should be noted that among children this fear and helplessness may present as disorganized or agitated behavior. The traumatic event may be re-experienced as an “instant replay” in the following ways by a child’s psyche: (a) they may engage in play where certain themes or aspects of the trauma are expressed; (b) it may be manifested as the appearance of frightening dreams without discernable content; and (c) the child may reenact specific aspects of the traumatic event (15, 16). Other manifestations of PTSD in this population have also been described but are beyond the scope of this focused debate (16, 17).

### Post-traumatic stress alters brain function and structure

It has been evident for over two decades that witnessing DV is capable of producing post-traumatic stress reactions among children present at the time of the violent acts (6, 16). It has been demonstrated that these children may develop serious adjustment and behavioral problems (18, 19). Given the evidence at hand, there is no doubt that DV can produce post-traumatic stress responses in children (16, 18, 20). Tribulations in early childhood have been shown to have serious consequences on mental health, and early life strain has been associated with cognitive difficulties such as poor academic accomplishments, lower intelligence quotients, as well as poor language skills, deficient memory, lack of inhibition, and inattention (21). These problems can persist into adolescence and adulthood (20). A history of witnessing DV or sexual victimization in childhood may increase the risk of subsequent marriage to an abusive mate (16), and childhood cumulative trauma (but not adulthood trauma) may predict the complexity of PTSD symptoms in adults (22).

The neurobiology of the brain is at the center of the problem at hand (6, 23, 24). There are compelling differences in neuroanatomy and cognitive function in affected children. These differences suggest the impact of maltreatment causes a crucial compromise of brain anatomy and function, including changes in structure, physiology, and signaling pathways (24–27). This is a critical concept to understand in this context.

There are two categories of maltreatment: (a) acts of commission and (b) acts of omission (22, 28). Both forms of maltreatment may lead to altered physiologic and neuroimaging characteristics (29–32). Acts of commission are acts that are intentionally intended whereas acts of omission involve failed care or neglect (33). Acts of commission include physical, sexual, and emotional abuse (33, 34). Physical abuse involves inflicting pain, bruising, scars, loss of mobility, burns and shaking. Sexual maltreatment includes sexual abuse, exploitation, or exposure to sexual acts (34). Emotional abuse encompasses repeated verbal abuse, demeaning and hurtful comments, rejection, and foul language (35–37). Acts of omission include witnessing verbal abuse or physical family violence. This category also includes neglect, lack of adequate food, clothing, shelter, and/or general care (36).

The evidence supporting the morphologic changes within the child’s brain became evident with the advances of neuroimaging in the 1990s. The advent of magnetic resonance imaging (MRI), functional magnetic resonance imaging (fMRI) positron emission tomography (PET) scan, single photon emission computed tomography (SPECT), magnetic resonance spectroscopy (MRS), and diffusion tensor imaging (DTI) have provided indisputable proof that the changes in brain anatomy and function do occur in the brains of maltreated children (see below). A functional imaging (fMRI) study, using continuous stimuli to respond or ignore the “Go/No–Go” task, assessed sustained attention and response inhibition (35, 38). Studies have concluded that adolescents with maltreatment-related PTSD showed relatively decreased activation of the middle frontal cortex and increased activation in the left medial frontal gyrus and the anterior cingulate gyrus (6, 25–27, 29, 32, 39–45).

The areas of the brain involved in neurobiological changes for children witnessing DV include the midbrain, the limbic system, cortex, corpus callosum, and cerebellum (27, 40–43). Briefly, their importance can be outlined as follows (46–51). The midbrain is the “relay point” for visual and auditory messages. The midbrain remains underdeveloped in these children and they become distracted easily and cannot pay close attention to tasks. They may not only have PTSD, but also attachment disorders and/or attention deficit hyperactivity disorder (ADHD). The limbic system (amygdala, hippocampus, hypothalamus) houses the primitive centers for emotion, survival, fear, anger, and pleasure, including sex. It is also important for memory information and storage, as well as gauging the magnitude of a response. There is little evidence, and some of it is conflicting, regarding anatomic changes in the hippocampus and amygdala associated with DV. However, witnessing DV may lead to hippocampal atrophy (as a late effect of maltreatment). The cortex is necessary for executive function and understanding consequences. The prefrontal cortex (PFC) and gray matter may have smaller volumes. This is noteworthy because the PFC has a role in functions that relate to mature adult behavior, including attention, inhibition, memory, motor control, motivation, emotion, expression of personality, and moderation of learned social behavior. On the other hand, there is evidence that young males who exhibit bad conduct and show little emotion, i.e., callousness, can present with increased gray matter in the medial orbitofrontal and anterior cingulate cortices, as well as increased gray matter volume and concentration in the temporal lobes bilaterally (52). This may be a sign of a problem with maturation of the cortex and with an affect on morality, empathy, and the ability to make sound decisions. This finding may or may not be related to violence or witnessing violence (i.e., a hereditary disorder). The corpus callosum is the largest white matter structure in the brain and connects the right and left cerebral hemispheres and facilitates their communication, thus allowing both sides of brain to communicate, i.e., auditory, visual, and cognitive messages. Its volume decreases in abuse or witnessing violence. Finally, the cerebellum is involved in emotion and cognitive development and balance. It has innumerable connections with the frontal lobes and is important to the frontocerebellar nexus that modulates behavior, and its volumes are decreased in youths who have experienced maltreatment. Smaller cerebellar volumes were associated with earlier onset of PTSD. Evidence from neuroimaging studies indicates a connection between PTSD, other anxiety disorders, and substance abuse. Individuals with PTSD abuse substances in an attempt to relieve their symptoms, with a worsening of traumatic memories during substance withdrawal (1, 47).

Childhood exposure to stress and trauma can also interfere with the sympathetic nervous system and the hypothalamic–pituitary–adrenal (HPA) axis. When a human is frightened, a rush of adrenalin (epinephrine) ensures that the body can instinctively prepare for a quick exit to safety or self-defense. This is mediated by two key systems: the sympathetic nervous system and the HPA axis (46, 49). The sympathetic nervous system makes neurotransmitters and hormones that provide persistent and chronic elevations of “pulse” hormones that lead to changes in brain structure. Hence, the hyperarousal and hypervigilance of PTSD may become chronic, despite resolution of the initiating experience (i.e., witnessing the DV) (25, 26, 28–30, 32, 49). The activation of the HPA axis allows the release of corticotropin releasing hormone (CRH). CRH then triggers release of adrenocorticotropic hormone (ACTH) that in turn stimulates adrenal cortisol secretion (49). Cortisol will activate mineralocorticoid and glucocorticoid receptors (GR), which can be found throughout the brain. GR are involved in transcription and expression of genes for immunity, metabolism, cognition, and brain development. The HPA axis is strongly influenced by social circumstances in childhood and is susceptible to mental and physical trauma (49). Children who witness violence may have elevated basal cortisol levels, whereas adults who were maltreated as children may exhibit low basal cortisol levels and elevated ACTH levels when stressed. Maltreated children, as they develop or are re-exposed to violence, re-regulate their psychobiological responses to chronic stress by down-regulating CRH receptors. This is an adaptation that allows only a minimal neuronal response to CRH-induced cell pathway disruption (49). High CRH levels occur in adults who have been exposed to violence as children, and this chronic elevation leads to arousal, anxiety, aggression, hypervigilance, general sympathetic nervous system stimulation, depression, and problems with eating and sex, i.e., symptoms of PTSD and depression (49).

Therefore, the brain has the ability to modify neural/hormonal function, and its resultant response is impaired in children who have witnessed DV (26, 29, 30, 32). In other words, such children demonstrate the following: increased depression, anxiety, more self-harm, a deficient ability to learn, poor concentration, and a generalized irritability. To further complicate the matter, there may be a genetic component to resilience under stress (53). Recent work has demonstrated that a functional polymorphism in the gene encoding the neurotransmitter-metabolizing enzyme monoamine oxidase A (MAOA) may moderate the effects of maltreatment; children with a genotype characterized by high levels of MAOA expression were less likely to develop antisocial problems (53). The DV triangle of witnessed abuse and its ensuing neurobiological changes, in the face of a disadvantaged genetic functional polymorphism, may be a set-up for accelerated failure as a person in a subset of these children, and a problem for society in general.

The impact on the community at large is of importance and concern (54); the effects on child witnesses of DV extend beyond the families and children. These children have impaired learning skills, poor school performance, poor life developmental skills, and lose their ability to self-regulate (4, 23, 54–56). As these children age, they will have different existential memories and respond in a different manner than they would have otherwise. Consequently, society may have difficulty preserving individual safety through an inability to decrease violence, while at the same time it has to support unproductive or underproductive members of society.

Cumulatively, these findings support the presence of neuro-biological-developmental alterations in children witnessing DV, their ensuing PTSD, and the impression that cumulative childhood trauma (and not adulthood trauma) may predict the overall symptom complexity in adults (22). Interventions before the age of seven result in the best outcomes (interventions in teenagers are not as effective), thus highlighting the need for early, aggressive, professional intercession during the elementary school years (6, 23). Early recognition of these distressed children would permit the activation of appropriate social support and treatment measures, including the child’s removal from an abusive environment (53). Fostering safety in the child’s environment and supporting the child’s family optimize their adult potential through normal neuroanatomic and biological development.

## Summary

A child witness of DV, where no intervention occurs, may develop PTSD that results in permanent changes to their personality as well as their ability to interact effectively in society as an adult. Throughout our paper we make associations, between witnessing DV and PTSD with accompanying neurobiological changes. We acknowledge the concepts of equifinality and multifinality could come into play in the psychological, physical, and social situations regarding the children in question (57). However, this does not deter us from our position in that the commission of DV in the presence of a child should become a stand-alone felony. Identification, screening, and intervention in regard to children who witness DV are of paramount importance. We should pursue local, state, and federal grant funding in support of the above-mentioned cause. Additionally, aggressive community outreach is necessary, and must include the business, academic, and healthcare sectors. Our children are true treasures and our best investment for the future of society.

## Author Contributions

Areti Tsavoussis, Stanislaw P. A. Stawicki, Nicoleta Stoicea, and Thomas J. Papadimos all equally contributed to the idea, writing, and editing of the manuscript and agree with its content.

## Conflict of Interest Statement

The authors declare that the research was conducted in the absence of any commercial or financial relationships that could be construed as a potential conflict of interest.

## Supplementary Material

The Supplementary Material for this article can be found online at http://www.frontiersin.org/Journal/10.3389/fpubh.2014.00178/abstract
