# Supplementary material for: Child-Witnessed Domestic Violence and its Adverse Effects on Brain Development: A Call for Societal Self-Examination and Awareness
Source: Front Public Health. 2014 Oct 10;2:178. doi: 10.3389/fpubh.2014.00178 (PMC4193214; doi:10.3389/fpubh.2014.00178)
Supplement: Supplementary file 1 [file Table_1.PDF]

Supplementary Table S1. DSM 5 PTSD Criteria[a]

Criterion A: stressor

The person was exposed to: death, threatened death, actual or threatened serious injury, or actual or threatened sexual violence, as follows, **(one required):**
1.Direct exposure.
2.Witnessing, in person.
3.Indirectly, by learning that a close relative or close friend was exposed to television, movies, or pictures.

Criterion B: intrusion symptoms

The traumatic event is persistently re-experienced in the following way(s), **(one required)**:
1.Recurrent, involuntary, and intrusive memories. Note: Children older than six may express this symptom in repetitive play.
2.Traumatic nightmares. Note: Children may have frightening dreams without content related to the trauma(s).
3.Dissociative reactions (e.g., flashbacks) which may occur on a continuum from brief episodes to complete loss of consciousness. Note: Children may reenact the event in play.
4.Intense or prolonged distress after exposure to traumatic reminders.
5.Marked physiologic reactivity after exposure to trauma-related stimuli.

Criterion C: avoidance

Persistent effortful avoidance of distressing trauma-related stimuli after the event, **(one required):**
1.Trauma-related thoughts or feelings.
2.Trauma-related external reminders (e.g., people, places, conversations, activities, objects, or situations).

trauma. If the event involved actual or threatened death, it must have been violent or accidental.
4.Repeated or extreme indirect exposure to aversive details of the event(s), usually in the course of professional duties (e.g., first responders, collecting body parts; professionals repeatedly exposed to details of child abuse). This does not include indirect non-professional exposure through electronic media,

Criterion D: negative alterations in cognitions and mood

Negative alterations in cognitions and mood that began or worsened after the traumatic event, **(two required)**:
1.Inability to recall key features of the traumatic event (usually dissociative amnesia; not due to head injury, alcohol, or drugs).
2.Persistent (and often distorted) negative beliefs and expectations about oneself or the world (e.g., "I am bad," "The world is completely dangerous").
3.Persistent distorted blame of self or others for causing the traumatic event or for resulting consequences.
4.Persistent negative trauma-related emotions (e.g., fear, horror, anger, guilt, or shame).
5.Markedly diminished interest in (pre-traumatic) significant activities.
6.Feeling alienated from others (e.g., detachment or estrangement).
7.Constricted affect: persistent inability to experience positive emotions.

Criterion E: alterations in arousal and reactivity

Trauma-related alterations in arousal and reactivity that began or worsened after the traumatic event, **(two required):**
1.Irritable or aggressive behavior
2.Self-destructive or reckless behavior
3.Hypervigilance
4.Exaggerated startle response
5.Problems in concentration
6.Sleep disturbance

Criterion F: duration

Persistence of symptoms (in Criteria B, C, D, and E) for more than one month.

Criterion G: functional significance

Significant symptom-related distress or functional impairment (e.g., social, occupational).

Criterion H: exclusion

Disturbance is not due to medication, substance use, or other illness.

Specify if: With dissociative symptoms.

In addition to meeting criteria for diagnosis, an individual experiences high levels of either of the following in reaction to trauma-related stimuli:
1.Depersonalization: experience of being an outside observer of or detached from oneself (e.g., feeling as if "this is not happening to me" or one were in a dream).
2.Derealization: experience of unreality, distance, or distortion (e.g., "things are not real").

Specify if: With delayed expression.

Full diagnosis is not met until at least six months after the trauma(s), although onset of symptoms may occur immediately.

References

[a]American Psychiatric Association. (2013) *Diagnostic and statistical manual of mental disorders*, (5th ed.). Washington, DC: Author.
